# The Physical Mechanism of Linear and Nonlinear Optical Properties of Nanographene-Induced Chiral Inversion

**DOI:** 10.3390/molecules29051053

**Published:** 2024-02-28

**Authors:** Zhiyuan Yang, Xinwen Gai, Yi Zou, Yongjian Jiang

**Affiliations:** 1 College of Science, Liaoning Petrochemical University, Fushun 113001, China; 15144700202@163.com (Z.Y.); gaixinwen1025@163.com (X.G.); 2 Liaoning Provincial Key Laboratory of Novel Micro-Nano Functional Materials, Fushun 113001, China

**Keywords:** nanographene, UV-Vis spectra, electron delocalization degree, electrical–magnetic interaction

## Abstract

Based on density functional theory (DFT) and wave function analysis, the ultraviolet and visible spectrophotometry (UV-Vis) spectra and Raman spectra of 1-meso and 1-rac obtained by the chiral separation of chiral nanographenes are theoretically investigated. The electron excitation properties of 1-meso and 1-rac are studied by means of transition density matrix (TDM) and charge density difference (CDD) diagrams. The intermolecular interaction is discussed based on an independent gradient model based on Hirshfeld partition (IGMH). The interaction of 1-meso and 1-rac with the external environment is studied using the electrostatic potential (ESP), and the electron delocalization degree of 1-meso and 1-rac is studied based on the magnetically induced current under the external magnetic field. Through the chiral separation of 1-rac, two enantiomers, 1-(P, P) and 1-(M, M), were obtained. The electrical–magnetic interaction of the molecule is revealed by analyzing the electron circular dichroism (ECD) spectra of 1-meso, 1-(P, P) and 1-(M, M), the transition electric dipole moment (TEDM) and the transition magnetic dipole moment (TMDM). It is found that 1-(P, P) and 1-(M, M) have opposite chiral properties due to the inversion of the structure.

## 1. Introduction

Graphene is a two-dimensional carbon nanomaterial composed of carbon atoms with sp^2^ hybrid orbitals in a hexagonal honeycomb lattice [1]. It is ultra thin, ultra light and super strong, and has many interesting properties, thus being known as the “king of new materials” [2,3]. Due to the hexagonal lattice structure formed by carbon atoms, graphene has two typical types of edges, namely, armchair edges and zigzag edges [4]. Graphene has attracted increasing attention due to its unique physical properties (such as strong mechanical strength, large surface area and good thermal, optical and electrical conductivity). The discovery of graphene has significantly accelerated research on the manufacture of low-cost electrode materials [5,6,7,8,9,10]. Graphene and its derivatives, including graphene oxide (GO) and reduced graphene oxide (r GO), are emerging as an important class of nanomaterials in the field of biosensors [11,12,13,14,15].

Since the discovery of graphene manufacturing methods by K. S. Novoselov et al. in 2003, the study of nanographene has become a major trend as the size of graphene devices reduces [16,17,18,19,20]. Nanographene is a type of graphene fragment with dimensions in the nanometer range. In recent years, with the development of nanographene chemistry, it has been increasingly applied in electronic devices, energy storage, sensing and other fields [21,22,23,24].

Chirality is widespread in nature. If an object is different from its mirror image, it is referred to as “chiral”, and its mirror image cannot coincide with the original object [25,26]. Chiral objects and their mirror images are called enantiomers. Chiral nanographenes with a high fluorescence quantum yield ($\phi_{F}$) and large asymmetry factor ($g_{\mathrm{lum}}$) are essential for the development of circularly polarized luminescence (CPL) materials. Wang at el. proposed a new strategy to extend the helical π of luminous groups with a high diameter $\phi_{F}$ to obtain chiral nanographenes with excellent luminous properties while maintaining the distribution of frontier molecular orbitals (FMOs). Using perylene as the parent core, the authors synthesized chiral nanographenes with an $\phi_{F}$ content of 93% and separated them by chiral HPLC to obtain 1-meso and 1-rac [27].

Besides experimental studies, there is a lack of theoretical work on 1-meso and 1-rac, particularly in the analysis and interpretation of their UV-Vis spectra, charge transfer properties and chiral mechanism, as well as other physical mechanisms. In this paper, the two chiral structures are introduced. Based on quantum chemical calculations and wave function analysis, the structure is theoretically studied by generating UV-Vis, ECD and Raman spectra, and the physical mechanism of light absorption is explained and combined with the visualization of the charge transfer process. This study provides a theoretical basis for the application of chiral nanographene structures in optoelectronics, linear optics and nonlinear optics.

## 2. Results

**Figure 1:**
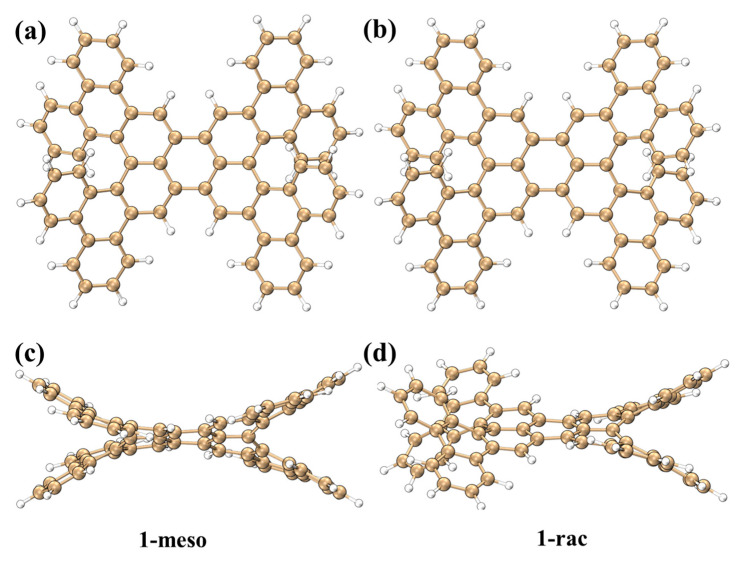
Structure diagram of 1-meso (**a**); structure diagram of 1-rac (**b**); structural side view of 1-meso (**c**); structural side view of 1-rac (**d**).

By taking perylene as the core, 1-meso and 1-rac were designed and synthesized as chiral nanographenes. The two enantiomers of chiral nanographenes were obtained through chiral separation. Their structures are presented in Figure 1a,b. The directions and angles of molecular torsion on both sides are different, as shown in Figure 1c,d. Figure S1 draws the IGMH diagram between the upper and lower segments of 1-meso and 1-rac at the torsion angle position. There are green isosurfaces in the close distance between the two intersecting benzene rings, suggesting the presence of interlayer interaction between the upper and lower layers. However, the electron density in the interaction region is very low. In this section, the optical properties, chirality mechanism and vibration modes of 1-meso and 1-rac will be investigated.

### 2.1. HOMO-LUMO Molecular Orbital Analysis

**Figure 2:**
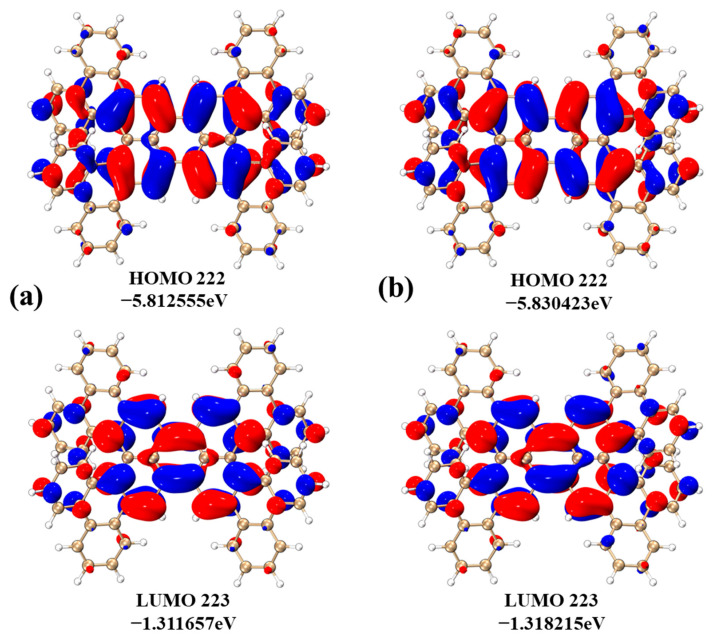
HOMO-LUMO molecular orbitals of 1meso (**a**) and 1-rac (**b**).

Figure 2a and Figure 2b, respectively, present the HOMO-LUMO molecular orbitals of 1-meso and 1-rac, where the positive and negative phases of the highest occupied molecular orbital (HOMO) and lowest unoccupied molecular orbital (LUMO) are shown in red and blue, respectively. The HOMO and LUMO energies of both 1-meso and 1-rac are depicted in Figure 2. It is worth noting that the HOMO-LUMO gap of 1-meso is measured at 2.535 eV, while that of 1-rac is 2.551 eV. The HOMO and LUMO energies indicate the ease with which a molecule can gain or lose electrons, and the HOMO-LUMO gap reflects the ability of electrons to transition from occupied to empty orbitals. A higher gap value indicates a lower probability of electron transition. Comparatively, 1-meso exhibits a greater tendency for excitation than 1-rac. By examining the molecular orbital diagrams, both the HOMO and LUMO diagrams demonstrate a similar distribution of electrons and holes, mainly concentrated on the central perylene. In addition, the transition from S_0_ to S_1_ represents a local excitation process.

### 2.2. The Physical Mechanism Analysis of UV-Vis Spectra

**Figure 3:**
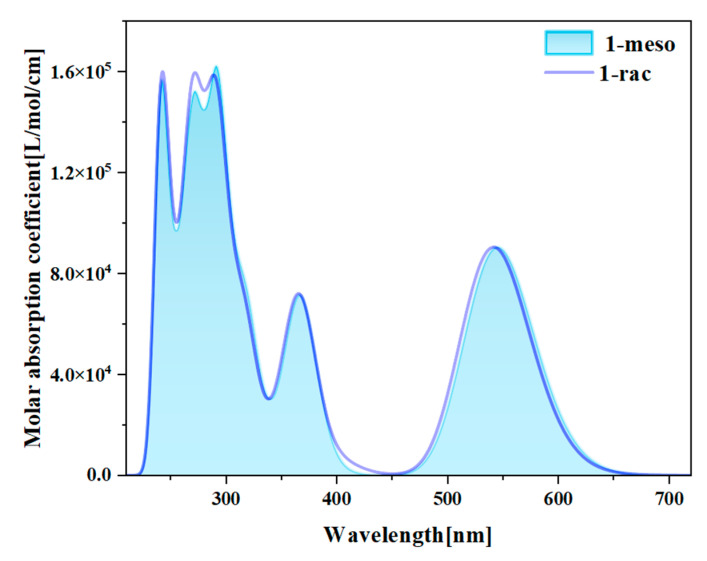
Combined absorption spectra of 1-meso and 1-rac.

We have plotted the UV-Vis spectra of 1-meso and 1-rac, as shown in Figure S2a,b. Figure 3 presents the combined UV-Vis spectra of 1-meso and 1-rac. The compounds 1-rac and 1-meso exhibit similar absorption spectra, with a slight redshift in the absorption spectra of 1-meso. This is consistent with the results of the HOMO-LUMO gap analysis. In the experiment, the maximum absorption values of the lowest energies of the compounds 1-rac and 1-meso are 538 nm and 539 nm, respectively, which is relatively small compared to the maximum absorption values of 545 nm and 546 nm of the lowest energies of 1-rac and 1-meso in the calculation of TD-DFT, reflecting the accuracy of the calculation [27]. The UV-Vis spectra show that the absorption spectra bands of 1-meso and 1-rac are mainly within 220–700 nm. They both have five absorption peaks. Due to their similar electronic structure, the photophysical properties in the ground state are not affected by the conformation.

### 2.3. Electronic Transition Properties

To further analyze the excitation properties of electron spectra, TDM and CDD maps of excited states where 1-meso and 1-rac contribute significantly to the absorption peaks are drawn. In the CDD diagram, the red isosurfaces represent electrons, and the blue isosurfaces represent holes. The expression for electrons and holes is as follows:

(1) $$\rho^{\mathrm{hole}}(r)=\rho_{\left(\mathrm{loc}\right)}^{\mathrm{hole}}(r)+\rho_{\left(\mathrm{cross}\right)}^{\mathrm{hole}}(r)$$

(2) $$\rho_{\left(\mathrm{loc}\right)}^{\mathrm{hole}}(r)=\sum_{i\to a}(w_{i}^{a}{)}^{2}\varphi_{i}\varphi_{i}-\sum_{i\leftarrow a}(w{’}_{i}^{a}{)}^{2}\varphi_{i}\varphi_{i}$$

(3) $$\rho_{\left(\mathrm{cross}\right)}^{\mathrm{hole}}(r)=\sum_{i\to a}\sum_{j\neq i\to a}w_{i}^{a}w_{j}^{a}\varphi_{i}\varphi_{j}-\sum_{i\leftarrow a}\sum_{j\neq i\leftarrow a}w{’}_{i}^{a}w{’}_{j}^{a}\varphi_{i}\varphi_{j}$$

(4) $$\rho^{\mathrm{ele}}(r)=\rho_{\left(\mathrm{loc}\right)}^{\mathrm{ele}}(r)+\rho_{\left(\mathrm{cross}\right)}^{\mathrm{ele}}(r)$$

(5) $$\rho_{\left(\mathrm{loc}\right)}^{\mathrm{ele}}(r)=\sum_{i\to a}(w_{i}^{a}{)}^{2}\varphi_{a}\varphi_{a}-\sum_{i\leftarrow a}(w{’}_{i}^{a}{)}^{2}\varphi_{a}\varphi_{a}$$

(6) $$\rho_{\left(\mathrm{cross}\right)}^{\mathrm{ele}}(r)=\sum_{i\to a}\sum_{i\to b\neq a}w_{i}^{a}w_{i}^{b}\varphi_{a}\varphi_{b}-\sum_{i\leftarrow a}\sum_{i\leftarrow b\neq a}w{’}_{i}^{a}w{’}_{i}^{b}\varphi_{a}\varphi_{b}$$

where r is the coordinate vector, φ is the orbital wave function, i or j is the occupied orbital label, and a or b is the empty orbital label. $\sum_{i\to a}$ represents each excitation configuration of the cycle, and $\sum_{i\leftarrow a}$ represents each de-excitation configuration of the cycle.

**Figure 4:**
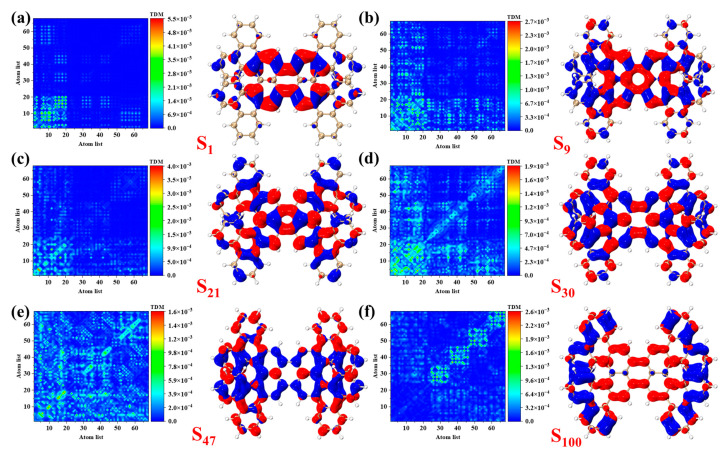
TDM and CDD diagrams of 1-meso under S_1_ (**a**), S_9_ (**b**), S_21_ (**c**), S_30_ (**d**), S_47_ (**e**), S_100_ (**f**). In CDD diagram, blue represents holes and red represents electrons. The isovalue is 0.0005.

Figure 4 shows the TDM and CDD diagrams of 1-meso. The absorption peak at 545 nm is contributed by S_1_, as shown in Figure S2a. The TDM and CDD of S_1_ are shown in Figure 4a. The transition density is mainly distributed in the lower left corner, and the electrons and holes are evenly distributed in the perylene in the middle of 1-meso. The absorption peak at 368 nm primarily originates from S_9_, which is characterized by the transition density being predominantly distributed on the left and lower sides. Local excitation occurs on the perylene molecule located in the center of 1-meso, with electrons from both sides transferring to the perylene center. The electronic transition mode observed under S_9_ involves local excitation accompanied by charge transfer, as depicted in Figure 4b. The absorption peak at 291 nm is primarily attributed to transitions occurring in S_21_ and S_30_, with the transition density primarily concentrated in the lower left corner of the diagonal, albeit with a small amount dispersed in other positions. Electrons and holes are evenly distributed across the perylene molecule, with a small presence in other regions. These characteristics of local excitation are depicted in Figure 4c,d. Similarly, the absorption peak at 273 nm is ascribed to S_47_, while the absorption peak at 241 nm is primarily contributed by S_100_. The electron transition mode in these two excited states primarily involves charge transfer excitation. Specifically, electrons in S_47_ transition from the middle of 1-meso to both the upper and lower sides, while electrons from both sides of 1-meso transfer to perylene in S_100_.

Figure S3 presents the TDM and CDD of 1-rac in various excited states. The excited states corresponding to the UV-Vis absorption peaks of 1-rac are highly similar to those of 1-meso molecules, except for the S_31_ and S_49_ excited states. A comparison of the TDM and CDD diagrams reveals nearly identical electronic transition properties, which can be attributed to their similar electronic structures.

### 2.4. ESP Analysis

**Figure 5:**
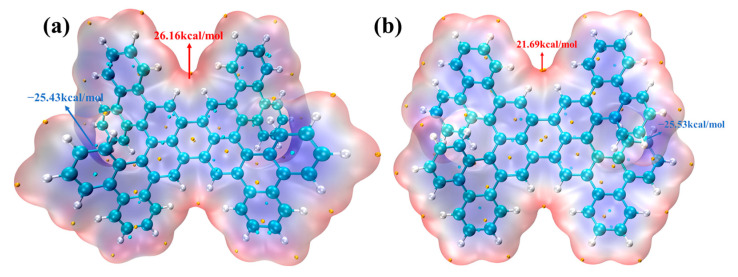
ESP isosurface diagram of 1-meso (**a**) and 1-rac (**b**).

In the ESP diagram of the molecular surface, the ESP value of different surface regions is represented by different colors, making the ESP distribution on the molecular surface clear. The value of the ESP reflects the ability of the molecule to interact with other molecules. Figure 5, respectively, shows the ESP of the molecular surface for 1-meso and 1-rac. Red indicates a positive ESP value, while blue indicates a negative ESP value. The yellow dots represent the maximum, and the blue dots represent the minimum. It can be observed that the ESP in the region where the C atom is located is negative, with the minimum value distributed near the C atom; in contrast, the ESP in the region where the H atom is located is positive, with the maximum value distributed above the H atom. The maximum value of 1-meso is significantly higher than that of 1-rac, while there is almost no difference in the minimum value. Consequently, the H atom corresponding to the maximum ESP of 1-meso is more prone to undergoing nucleophilic reactions.

### 2.5. Response to External Magnetic Field and Magnetic Induced Current Density

**Figure 6:**
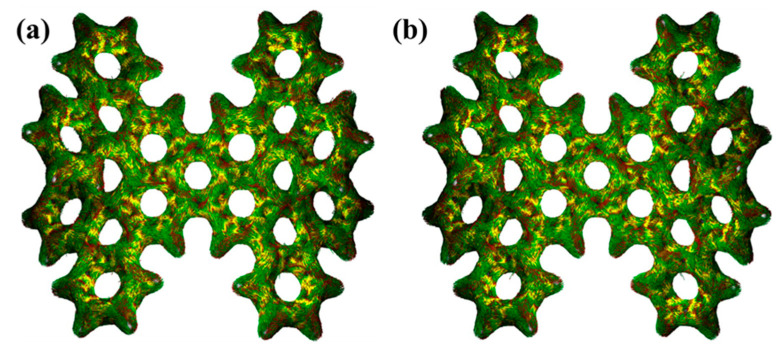
Magnetic induced current densities of 1-meso (**a**) and 1-rac (**b**).

If the electrons in a molecular system have strong delocalization, a significant induced ring current will be generated when a magnetic field is applied. Therefore, we studied the induced currents of 1-meso and 1-rac induced by an external magnetic field, which is perpendicular to the molecular plane from inside to outside, as shown in Figure 6, Figures S4 and S5 (in the Supplementary Materials). The induced currents of 1-meso and 1-rac are clearly circular, and the red circular arrow indicates the direction of the system’s circular current. It can be seen from the figure that the current isosurface in the yellow region is mainly distributed on the inner side of the benzene ring. The ring current in each benzene ring is clockwise, and a clockwise ring current is also formed at the edge of the molecule. This indicates that 1-meso and 1-rac have strong electron dissociation.

### 2.6. Raman Spectral Analysis

The Raman spectra are a type of scattering spectra, where the horizontal axis represents the frequency of the scattered light in relation to the incident light, and the vertical axis indicates the intensity of the scattered light. The Raman activity is a unique property of each vibration mode. The Raman spectrum can reflect the characteristic information of the molecular structure of substances, and is a unique property in the study of molecular chemical structure and chemical composition analysis.

**Figure 7:**
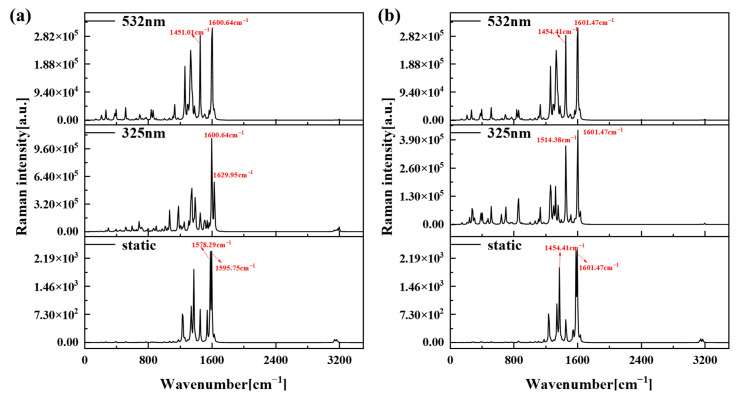
Raman and resonance Raman spectra of 1-meso (**a**) and 1-rac (**b**).

In the UV-Vis spectra, the most prominent absorption peaks for the 1-meso and 1-rac conformers within the ultraviolet wavelength range are observed at 291 nm, while the most intense absorption peak within the visible light band occurs at 545 nm. Therefore, we select two laser wavelengths (325 nm and 532 nm) that closely match these peaks to irradiate the respective structures, facilitating the acquisition of their resonance Raman spectra. Figure 7 shows the Raman and resonance Raman spectra of 1-meso (a) and 1-rac (b). It can be seen from Figure 7 that the resonance Raman spectra of 1-meso and 1-rac after laser irradiation have the same Raman peak wave number as the static Raman spectra, and the strongest Raman peak in both the static Raman spectra and the resonance Raman spectra is located near 1600 cm^−1^. However, the Raman intensity is significantly enhanced, indicating that both wavelengths of laser can significantly improve the Raman intensity of 1-meso and 1-rac by about two orders of magnitude.

**Figure 8:**
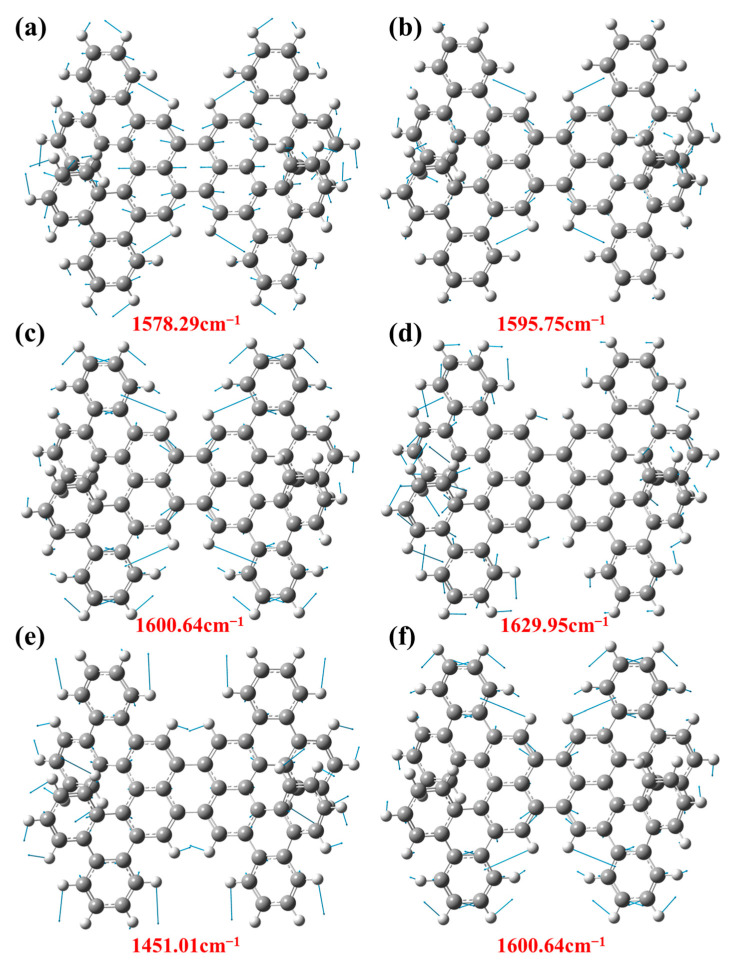
(**a**) The Vibration mode at position 1578.29 cm^−1^. (**b**) The vibration mode at position 1595.75 cm^−1^. (**c**) The vibration mode at 1600.64 cm^−1^ positions under laser irradiation at 325 nm. (**d**) The vibration mode at 1629.95 cm^−1^ positions under 325 nm laser irradiation. (**e**) The vibration mode at 1451.01 cm^−1^ positions under 532 nm laser irradiation. (**f**) The vibration mode at 1600.64 cm^−1^ positions under 532 nm laser irradiation.

Then, the vibration attribution of 1-meso and 1-rac Raman characteristic peaks are analyzed and explained, respectively. With different frequencies, the vibration modes of the molecule are also different. In the vibration mode diagram, the direction of the vector arrow indicates the main direction of the atomic vibration, and the length of the arrow indicates the intensity of the vibration, which is the energy involved in the vibration. Figure 8 shows the vibration modes corresponding to 1-meso in different bands. The vibration mode at 1578.29 cm^−1^ corresponds with the contraction of the central benzene ring and the vibration of the surrounding hydrogen atoms. On the contrary, the vibration mode at 1595.75 cm^−1^ represents the lateral vibration of hydrogen atoms, as shown in Figure 8a,b. Figure 8c,d depict the vibration modes observed at the prominent peak position of 1-meso in the 325 nm resonance Raman spectra, where the vibration mode at 1600.64 cm^−1^ signifies the vibration of hydrogen atoms and the stretching vibration of the carbon atoms in the benzene ring. Additionally, the vibration mode at 1629.95 cm^−1^ indicates the lateral oscillation of H atoms. Figure 8e,f display the vibration modes of 1-meso at the position of the prominent peak in the 532 nm resonance Raman spectra. The corresponding vibration patterns at the higher peaks are attributed to the vibration of the H atoms. Notably, the vibration mode at 1600.64 cm^−1^ corresponds to the same position under 325 nm laser irradiation.

**Figure 9:**
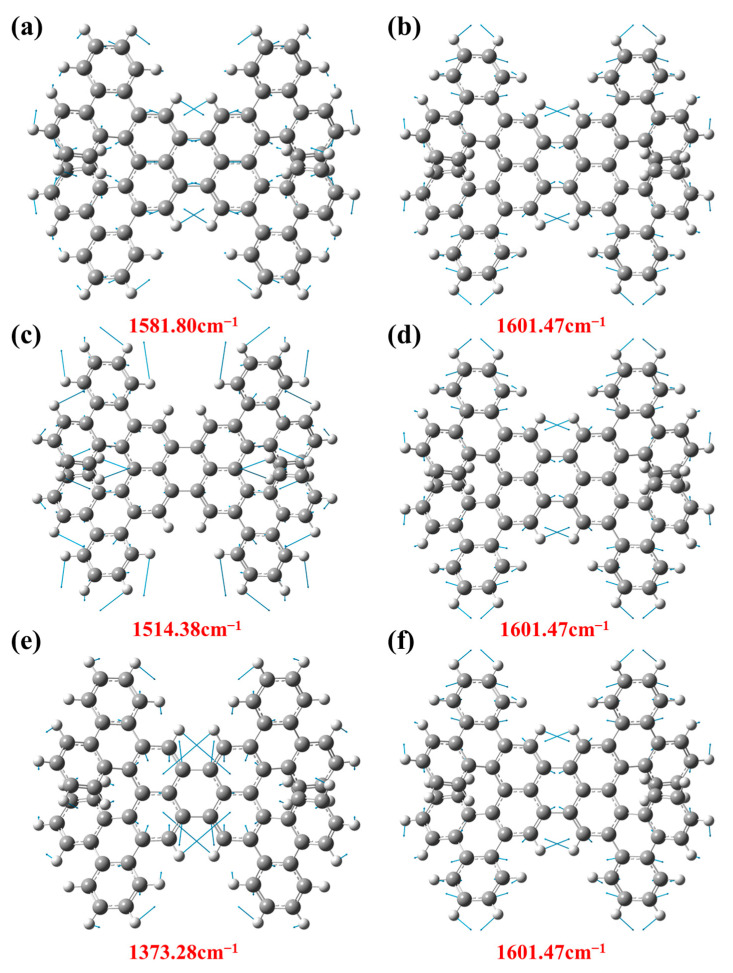
(**a**) The Vibration mode at position 1581.80 cm^−1^. (**b**) The vibration mode at position 1601.47 cm^−1^. (**c**) The vibration mode at 1514.38 cm^−1^ positions under laser irradiation at 325 nm. (**d**) The vibration mode at 1601.47 cm^−1^ positions under 325 nm laser irradiation. (**e**) The vibration mode at 1373.28 cm^−1^ positions under 532 nm laser irradiation. (**f**) The vibration mode at 1601.47 cm^−1^ positions under 532 nm laser irradiation.

Figure 9a,b depict the vibration mode of 1-rac observed at the peak position in the static Raman spectra. Both the peaks at 1581.80 cm^−1^ and 1601.47 cm^−1^ exhibit similar vibration patterns, which are attributed to the movement of C and H atoms. Figure 9c,d illustrate the vibration mode of 1-rac corresponding to the prominent peak in the 325nm resonance Raman spectra. Specifically, the vibration observed at 1514.38 cm^−1^ is related to the motion of C atoms and H atoms on both sides, excluding those associated with the intermediate perylene. In Figure 9e,f, the vibration mode of 1-rac is observed at the position of the strong peak in the 532 nm resonance Raman spectra. Here, the vibration observed at 1373.28 cm^−1^ is associated with the stretching of the central benzene ring and the lateral vibrations of additional H atoms. Notably, the vibration pattern observed at 1601.47 cm^−1^ remains consistent under the irradiation of three different lasers. Due to the certain symmetry of the molecules, their vibration patterns are also symmetrically stretched.

### 2.7. ECD Spectra and Transition Electric\Magnetic Dipole Moment

**Figure 10:**
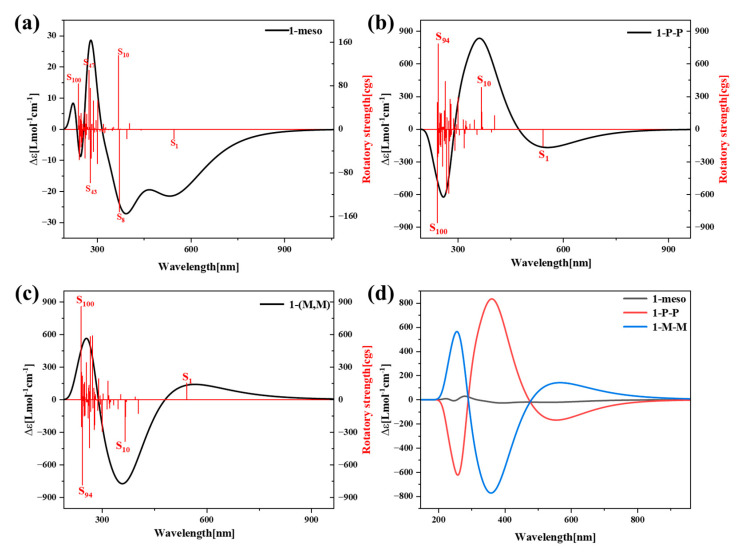
ECD spectra of 1-meso (**a**), 1-(P, P) (**b**), 1-(M, M) (**c**) and their combination diagram (**d**).

Ji-Kun Li et al. experimentally obtained two enantiomers of 1-(P, P) and 1-(M, M) by the chiral separation of 1-rac at room temperature [27], as shown in Figure S6. Therefore, we judge the chirality of 1-meso and 1-rac enantiomers by calculating their electron circular dichroism, as shown in Figure 10.

ECD can effectively characterize the chirality of chromophores in molecules. Theoretically, the strength of ECD can be defined as

(7) $$\propto \langle \varphi_{j}|\mu_{e}|\varphi_{i}\rangle \langle \varphi_{j}|\mu_{m}|\varphi_{i}\rangle B$$

$\mu_{e}$ represents the TEDM and $\mu_{m}$ represents the TMDM. Here, the first term in the absolute value is light absorption and the second term is circular dichroism. The dominant ECD spectra are the tensor products of the TEDM and TMDM. Figure 10a shows the ECD spectra of 1-meso, while Figure 10b,c present the ECD spectra of two enantiomers of 1-rac. Figure 10d is their combined diagram. It can be observed from the figure that the chirality of 1-meso is weak, the chirality of the two enantiomers of 1-rac are strong, and the images of 1-(P, P) and 1-(M, M) are symmetric and have opposite Cotton effects.

**Figure 11:**
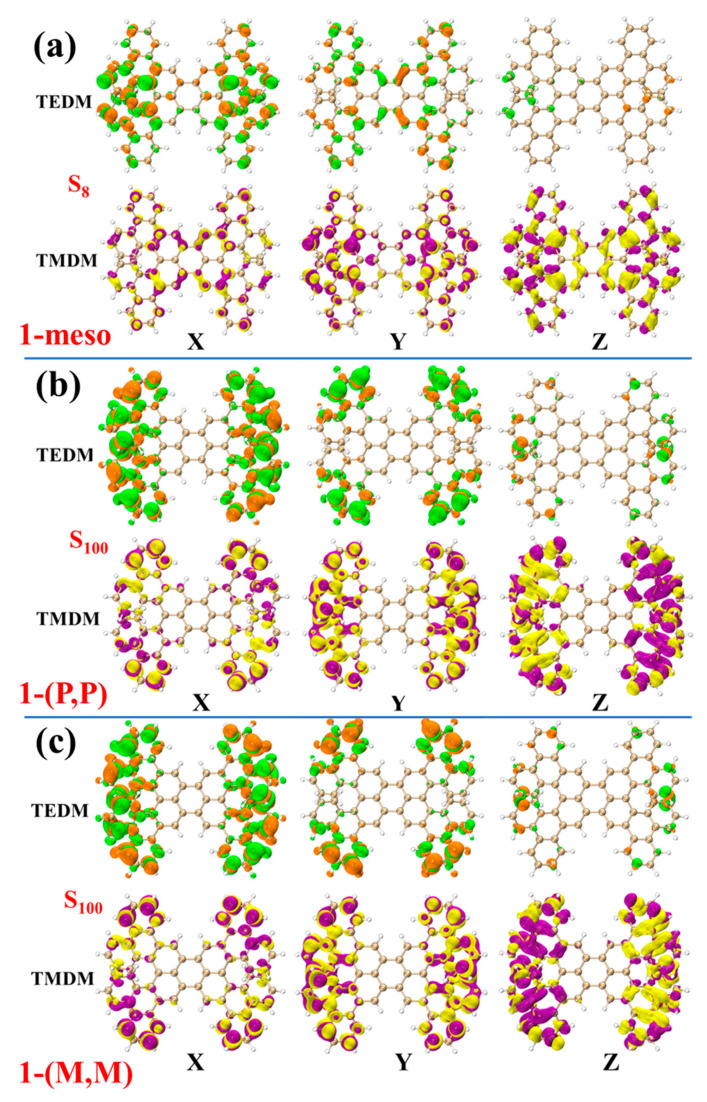
1-meso and S_8_ (**a**), 1-(P, P) and S_100_ (**b**) and 1-(M, M) and S_100_ (**c**) are TEDMs\TMDMs in different directions. The green (orange) isosurfaces represent the positive (negative) TEDM, and yellow (purple) isosurfaces represent the positive (negative) TMDM. X, Y, Z represent the direction.

Next, the properties of these three structural chirality were analyzed by studying the density of the TEDM\TMDM in different directions. As shown in Figure 10a, 1-meso has a positive rotatory strength in S_10_, S_46_ and S_100_, and a negative rotatory strength in S_1_, S_8_ and S_43_. The TEDM density of the six excited states successively along the X, Y and Z directions (Figure 11a and Figures S7–S11 in the Supplementary Materials). The TMDM density increases in all three directions, exhibiting an alternating distribution of positive and negative regions. Moreover, both the positive and negative isosurfaces of the TEDM and TMDM are predominantly distributed on the left and right sides of the molecule. Notably, the distributions of the TEDM and TMDM for states S_47_ and S_100_ are similar, with the positive and negative TMDM densities in the Z direction clearly separated. This indicates that the chiral effects of states S_47_ and S_100_ have a similar nature. In structure 1-(P, P), the rotatory strength of states S_10_ and S_94_ is positive, whereas that of states S_1_ and S_100_ is negative. Conversely, in 1-(M, M), the rotatory strength of S_10_ and S_94_ is negative, while that of S_1_ and S_100_ is positive. It is worth nothing that within the same excited state, the positive and negative isosurfaces of 1-(M, M) for both the TEDM and TMDM along the X, Y and Z directions are nearly opposite to the positive and negative isosurfaces of 1-(P, P); refer to Figure 11b,c and Figures S12–S17 in the Supplementary Materials. This discrepancy is due to the inherent symmetry between the chirality of 1-(P, P) and 1-(M, M). It is remarkable that the TEDM and TMDM densities of the three structures are mainly distributed on the left and right sides of the structure, rather than on perylene, indicating that the chirality is mainly attributed to the asymmetry on both sides of the structure.

Table 1 shows the TEDM and TMEM components and their eigenvalues of the excited states at X, Y and Z of 1-meso, 1-(P, P) and 1-(M, M), which make the highest contribution to the ECD spectra. The absolute value of the molecular tensor product represents the light excitation intensity, and the greater the absolute value, the stronger the excitation intensity. From the table, it can be seen that when the absolute value of the 1-meso tensor product is low, 1-(P, P) and 1-(M, M) are relatively high. Thus, the two structures obtained from 1-rac by chiral separation have strong chirality. The intensity and direction of each ECD spectra in the table were completely matched. The results show that the TEDM\TMDM is complete and self-consistent in inferring the chiral mechanism.

## 3. Calculation Method

In this research, we constructed the 1-meso and 1-rac models using GaussView 6.0.16 software [28] and Gaussian 16 (A.03) program [29]. The optimization of the structures was carried out based on the density functional theory (DFT) [30] calculation method, utilizing the B3LYP [31] functional and 6–31g(d) [32] basis set, along with DFT-D3 [33] correction. Subsequently, the B3LYP [34,35] function set and 6–31g(d) base set were used for the electronic excitation calculation using the TD-DFT calculation method, followed by the visualization and analysis of the excitation results using Multiwfn [36]. The UV-Vis spectra, ECD spectra, TMD and Raman spectra of the system were generated using Origin 2022 software [37]. Furthermore, employing the VMD program [38], we plotted the CDD diagram, TEDM, TMDM, IGMH and ESP of the system [39,40,41]. Additionally, the vibration modes of 1-meso and 1-rac were depicted using GaussView, while the magnetic induced current density was illustrated using the AICD program [42].

## 4. Conclusions

In summary, we employ quantum chemistry calculations and wave function analysis to study the HOMO and LUMO molecular orbitals, UV-Vis and Raman spectra, and the ESP and magnetic induced current density of 1-meso and 1-rac. 1-(M, M) and 1-(P, P) are obtained through the chiral separation of 1-rac, and their chiral mechanisms are investigated. The HOMO and LUMO diagrams both exhibit a similar distribution of electrons and holes, primarily concentrated around the central perylene, where S_0_→S_1_ transitions represent local excitations. The TDM and CDD plots in the spectral analysis reflect the linear optical properties of the 1-meso and 1-rac. First, the UV-Vis spectra shows that 1-meso and 1-rac have similar electronic properties, resulting in consistent electronic excitation properties. The form of electron excitation is valence shell excitation, comprising local excitation and charge transfer excitation. The IGMH analysis reveals a weak interaction between the benzene rings. The ESP maxima of 1-meso and 1-rac are evidently different, with the maximum of 1-meso being significantly higher than that of 1-rac. The magnetic induced current density demonstrates strong electron delocalization in both 1-meso and 1-rac. Raman spectra analysis shows that lasers can significantly enhance the Raman intensity of 1-meso and 1-rac. Furthermore, chiral separation is employed to divide 1-rac into 1-(M, M) and 1-(P, P) structures. These resulting structures are mirror images of each other, exhibiting a stronger level of chirality compared to 1-meso. The analysis of the TEDM and TMDM reveals that the positive and negative phases of 1-(M, M) and 1-(P, P) are opposite in the X, Y and Z directions, indicating the presence of chiral symmetry between the two. This research provides theoretical insights into the photophysical properties of other chiral nanographene materials, potentially guiding future studies in this field.

## Figures and Tables

**Table 1:** The value of the transition electric/magnetic dipole moment and the eigenvalue of their tensor product.

		X	Y	Z	Eigenvalue
1-meso	TEDM	0.0000	−0.4417	0.1305	−0.6414
S_8_	TMDM	0.0000	−0.6016	2.8786	
1-(P,P)	TEDM	−0.0003	2.4479	−0.0000	−3.6456
S_100_	TMDM	−0.0001	1.4893	0.0001	
1-(M,M)	TEDM	−0.0005	−2.4478	0.0001	3.6450
S_100_	TMDM	0.0002	1.4891	0.0002	

## Data Availability

Data are contained within the article and Supplementary Materials.

## Supplementary Materials

The following supporting information can be downloaded at: https://www.mdpi.com/article/10.3390/molecules29051053/s1, Figure S1: IGMH isosurfaces of 1-meso and 1-rac (a, b), and their side views (c, d). Figure S2: UV-vis spectra of 1-meso (a) and 1-rac (b). Figure S3: TDM and CDD diagrams of 1-rac under S_1_ (a), S_9_ (b), S_21_ (c), S_31_ (d), S_49_ (e), S_100_ (f). In CDD diagram, blue represents holes and red represents electrons. The isovalue is 0.0005. Figure S4: Magnetic induced current density of 1-meso. Figure S5: Magnetic induced current density of 1-rac. Figure S6: Structure diagram of 1- (M, M) and 1- (P, P). Figure S7: TEDM and TMDM of 1-meso in S_1_ excited state. Figure S8: TEDM and TMDM of 1-meso in S_10_ excited state. Figure S9: TEDM and TMDM of 1-meso in S_43_ excited state. Figure S10: TEDM and TMDM of 1-meso in S_47_ excited state. Figure S11: TEDM and TMDM of 1-meso in S_100_ excited state. Figure S12: TEDM and TMDM of 1- (P, P) in S_1_ excited state. Figure S13: TEDM and TMDM of 1- (P, P) in S_10_ excited state. Figure S14: TEDM and TMDM of 1- (P, P) in S_94_ excited state. Figure S15: TEDM and TMDM of 1- (M, M) in S_1_ excited state. Figure S16: TEDM and TMDM of 1- (M, M) in S_10_ excited state. Figure S17: TEDM and TMDM of 1- (M, M) in S_94_ excited state.
