# Supplementary material for: The Physical Mechanism of Linear and Nonlinear Optical Properties of Nanographene-Induced Chiral Inversion
Source: Molecules. 2024 Feb 28;29(5):1053. doi: 10.3390/molecules29051053 (PMC10934395; doi:10.3390/molecules29051053)
Supplement: Supplementary file 1 [file molecules-29-01053-s001.zip › molecules-2831607-supplementary.pdf]

Supporting information

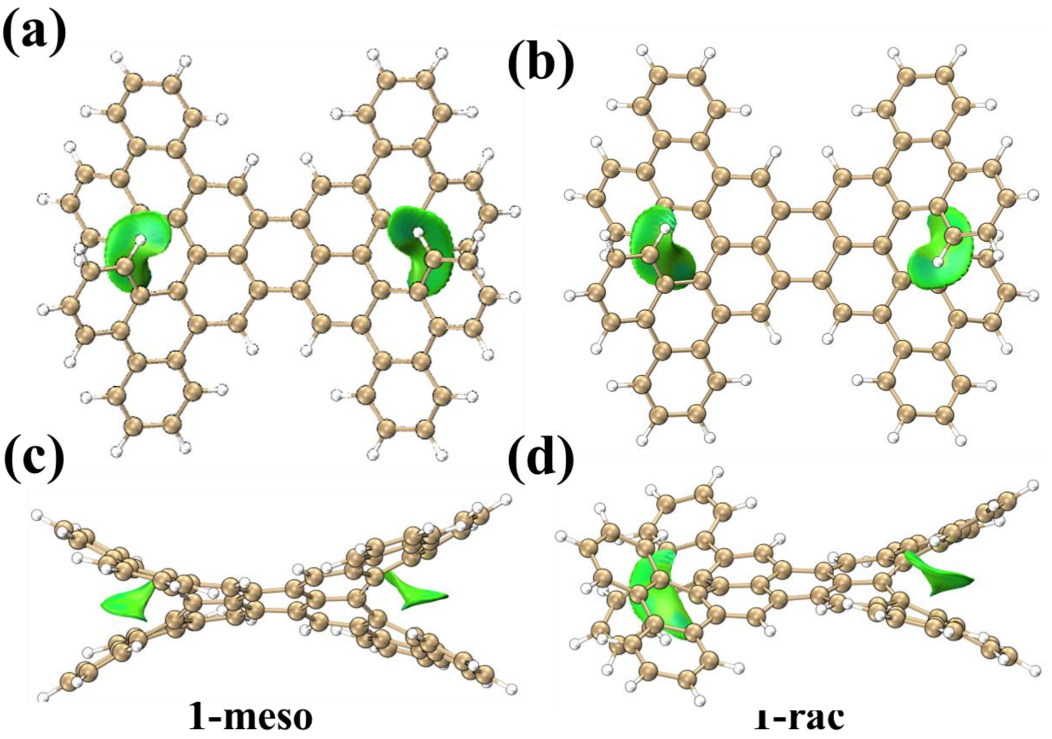

Figure S1. IGMH isosurfaces of 1-meso and 1-rac (a, b), and their side views (c, d).

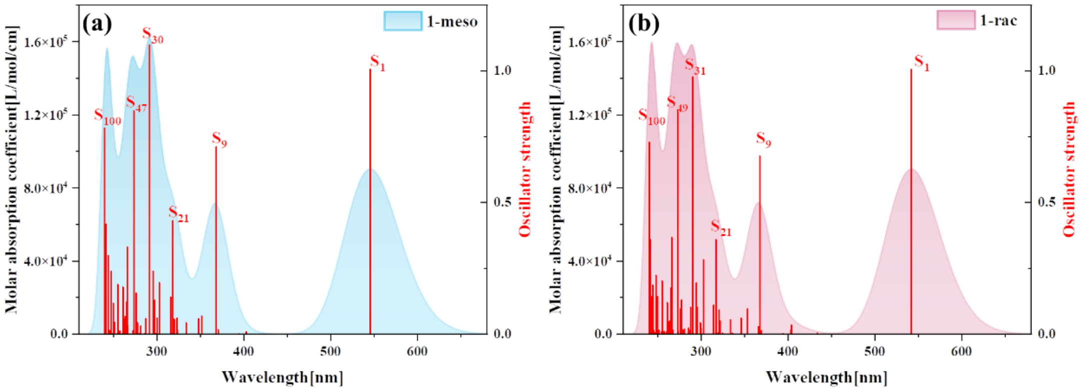

Figure S2. UV-vis spectra of 1-meso (a) and 1-rac (b).

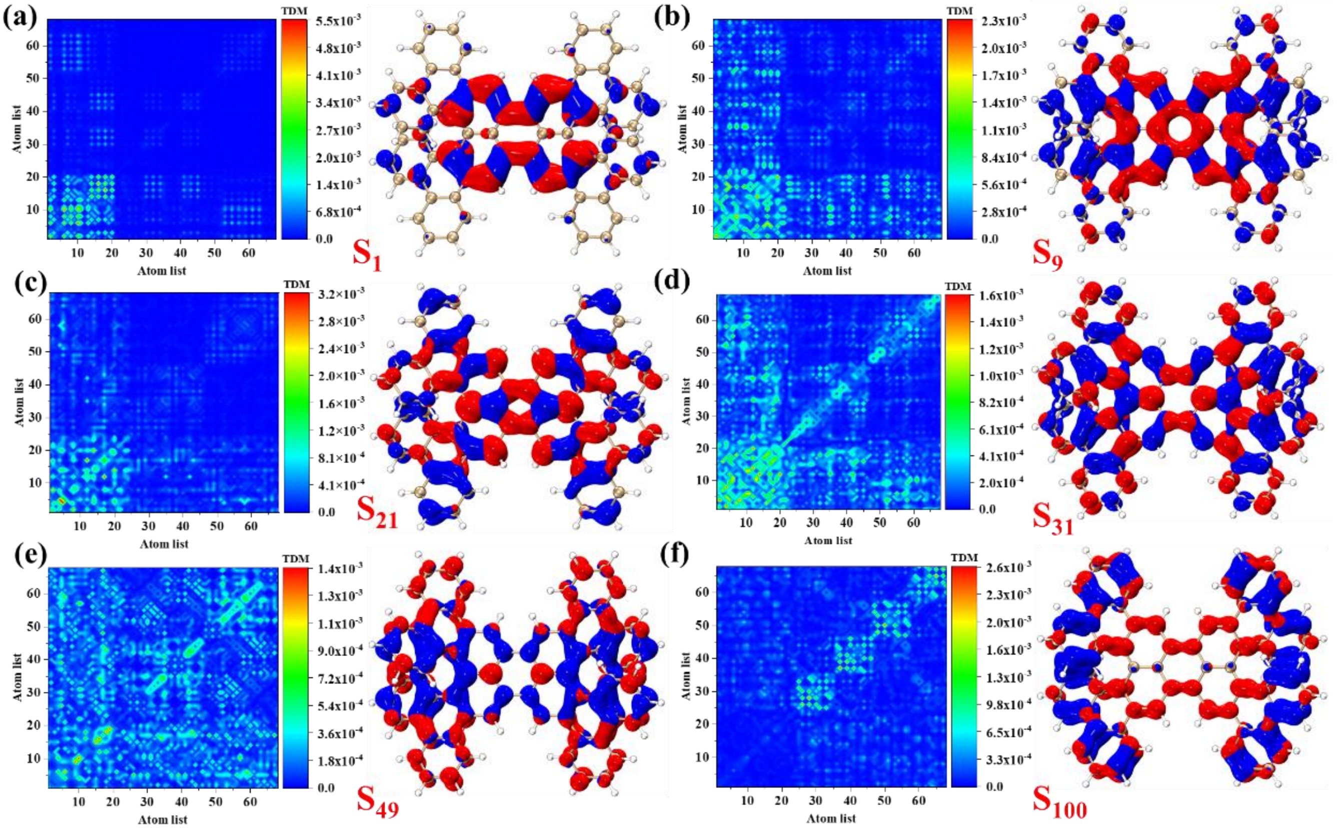

Figure S3. TDM and CDD diagrams of 1-rac under S_1 (a), S_9 (b), S_{21} (c), S_{31} (d), S_{49} (e), S_{100} (f). In CDD diagram, blue represents holes and red represents electrons. The isovalue is 0.0005.

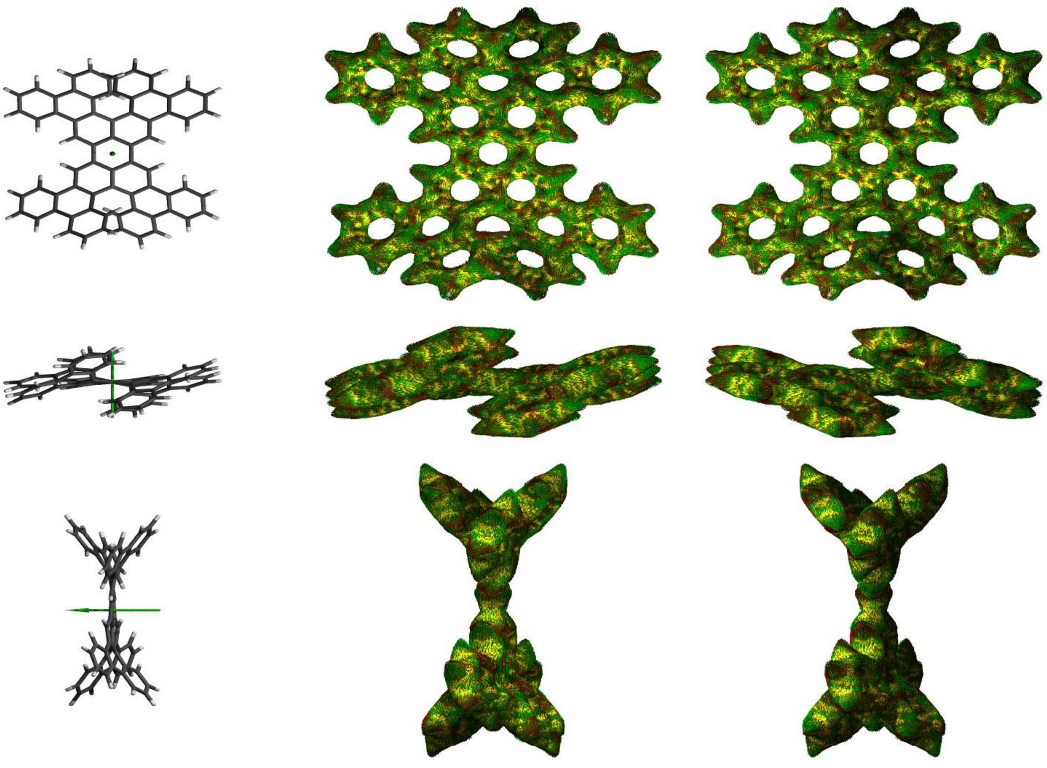

Figure S4. Magnetic induced current density of 1-meso.

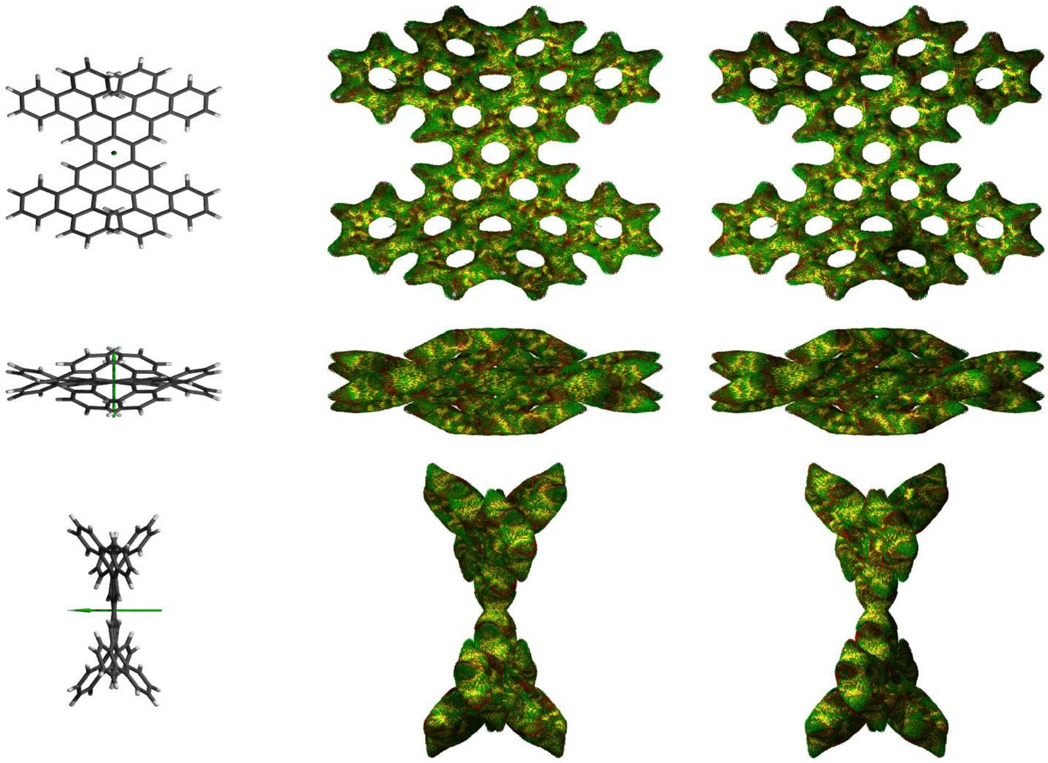

Figure S5. Magnetic induced current density of 1-rac.

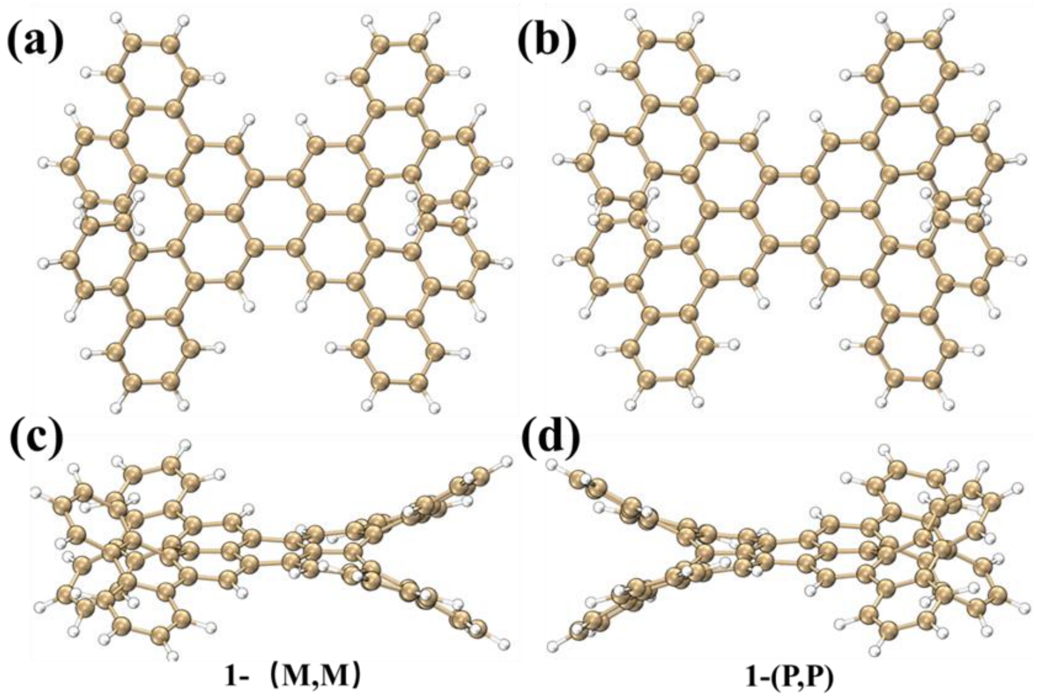

Figure S6. Structure diagram of 1- (M, M) and 1- (P, P).

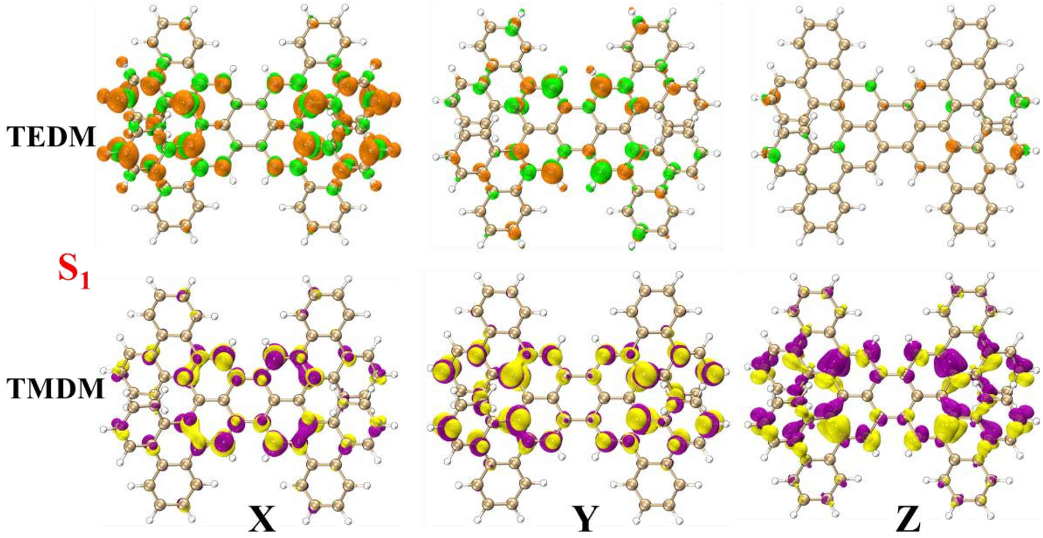

Figure S7. TEDM and TMDM of 1-meso in S_1 excited state.

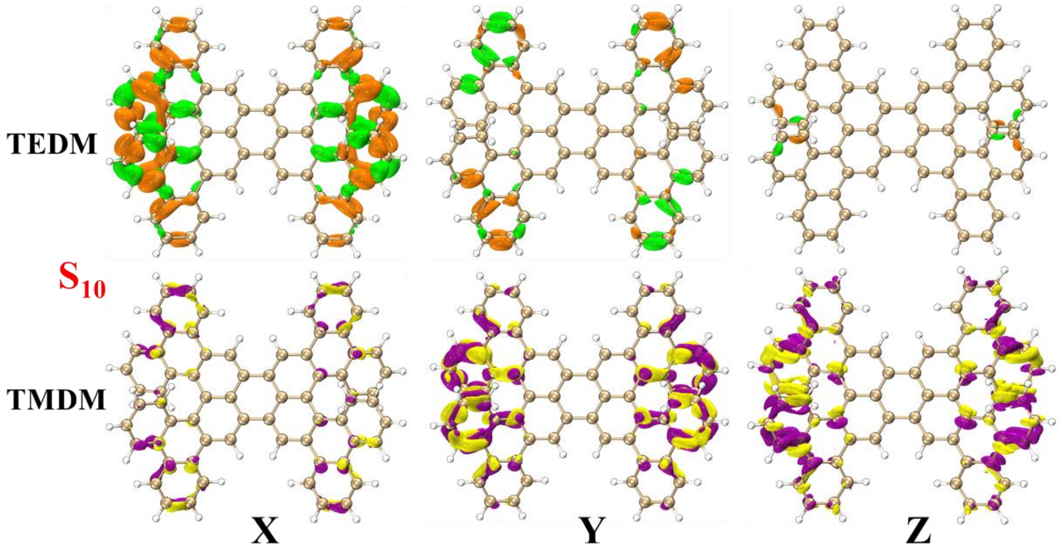

Figure S8. TEDM and TMDM of 1-meso in S_{10} excited state.

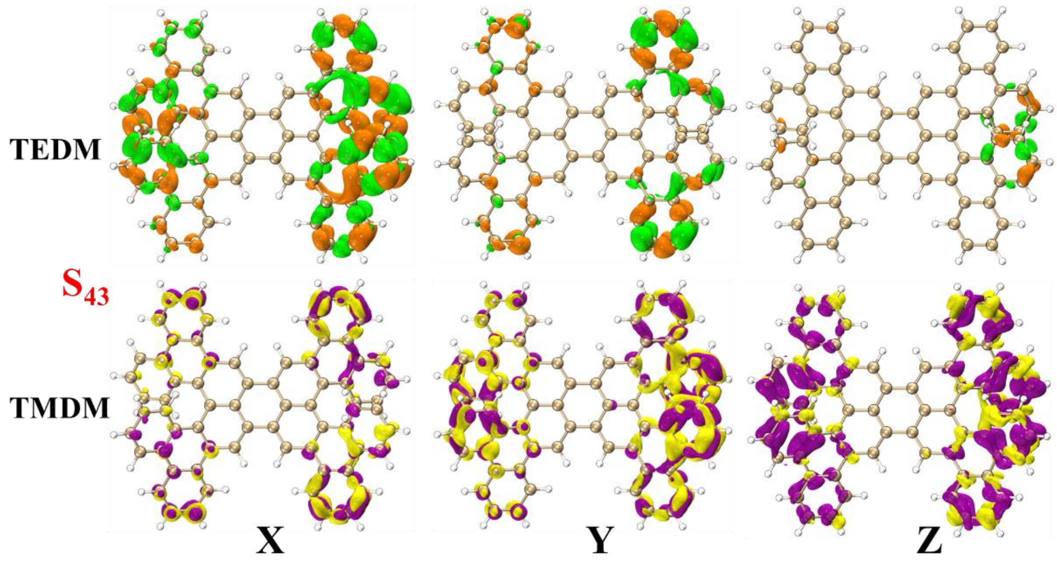

Figure S9. TEDM and TMDM of 1-meso in S_{43} excited state.

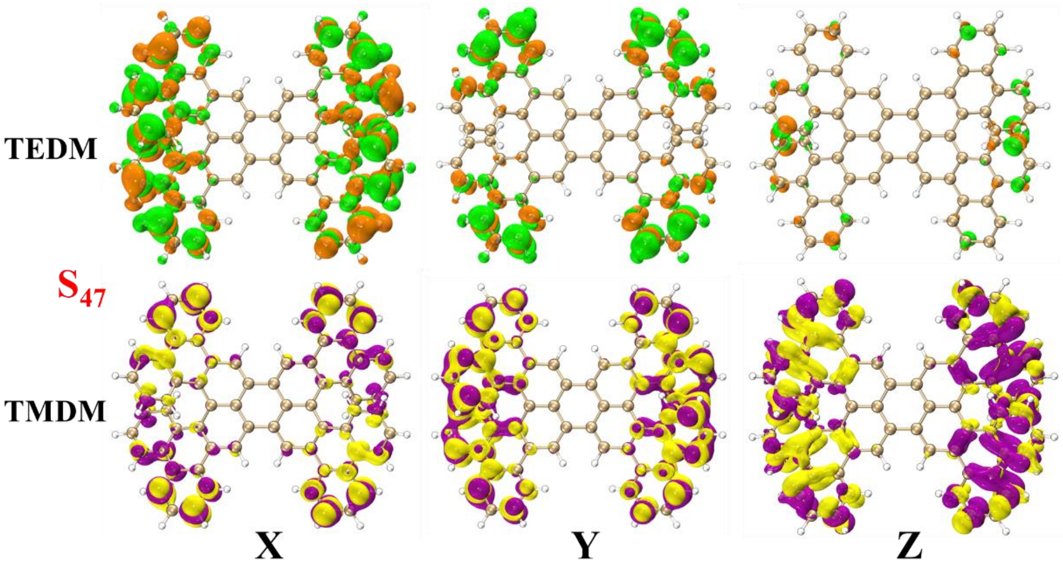

Figure S10. TEDM and TMDM of 1-meso in S_{47} excited state.

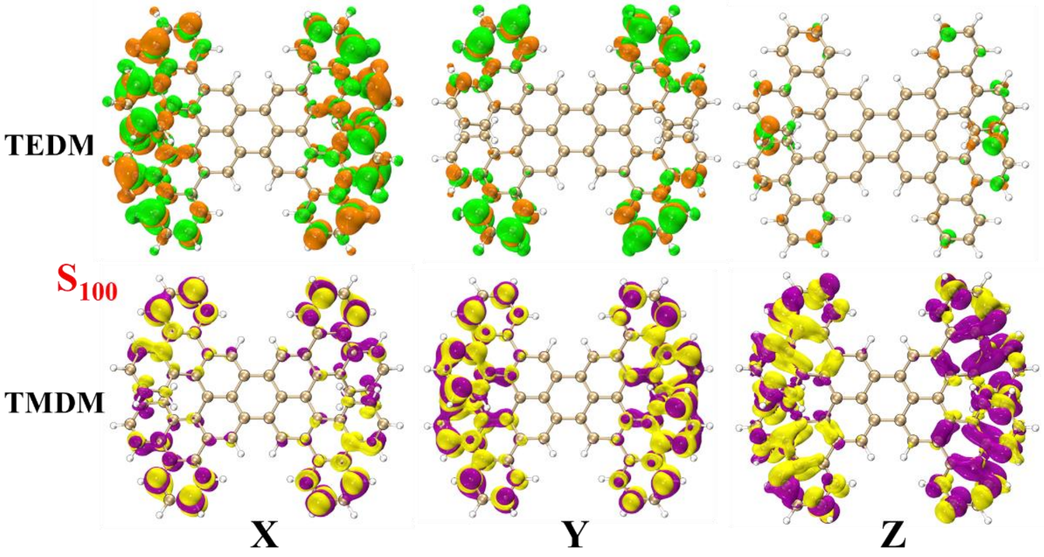

Figure S11. TEDM and TMDM of 1-meso in S_{100} excited state.

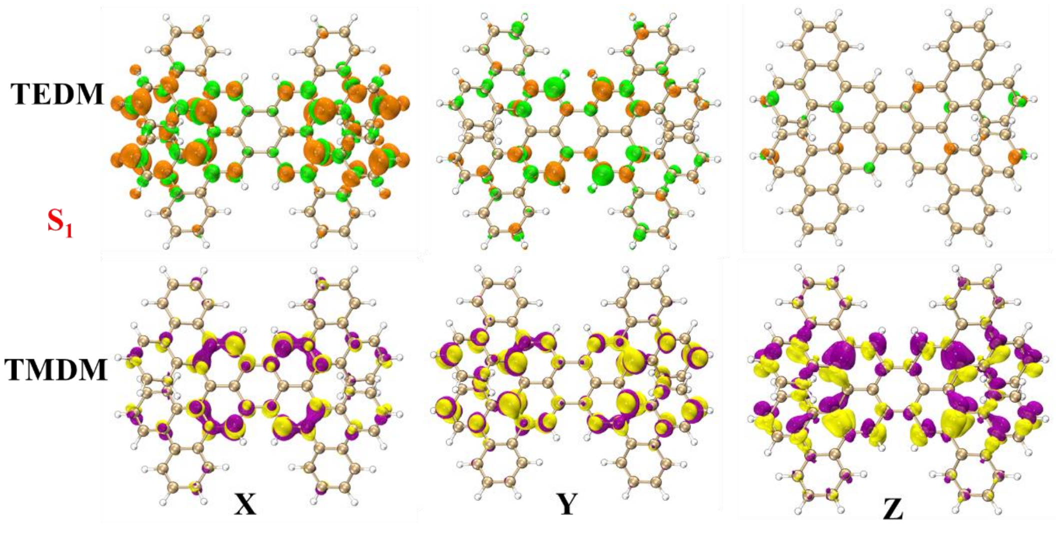

Figure S12. TEDM and TMDM of 1- (P, P) in S_1 excited state.

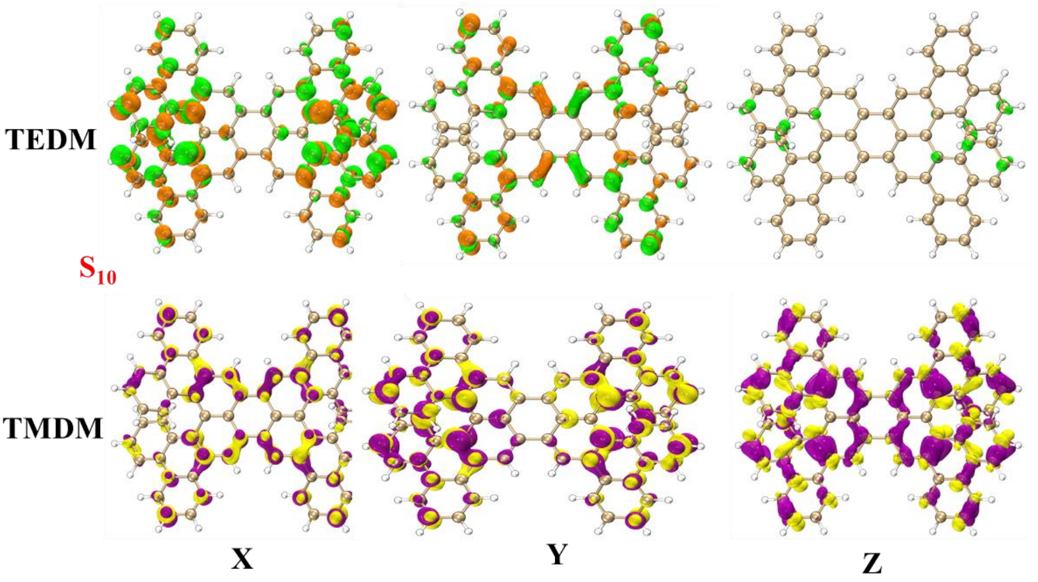

Figure S13. TEDM and TMDM of 1- (P, P) in S_{10} excited state.

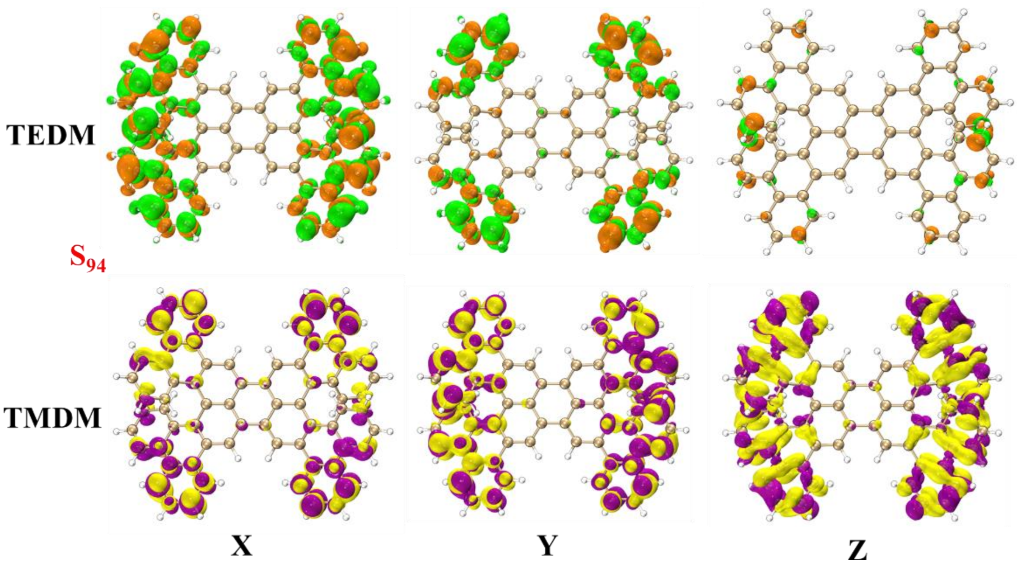

Figure S14. TEDM and TMDM of 1- (P, P) in S_{94} excited state.

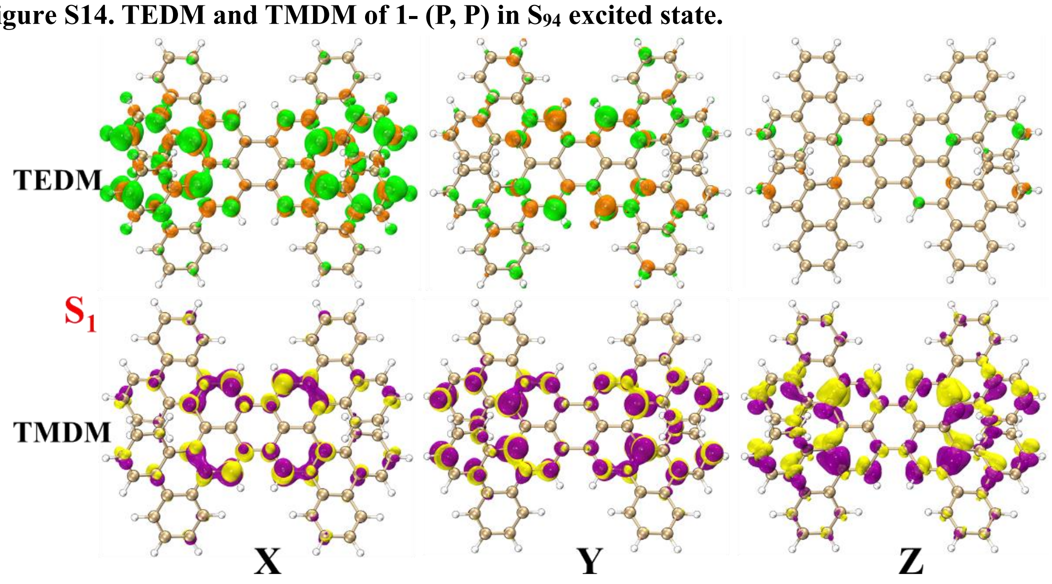

Figure S15. TEDM and TMDM of 1- (M, M) in S_1 excited state.

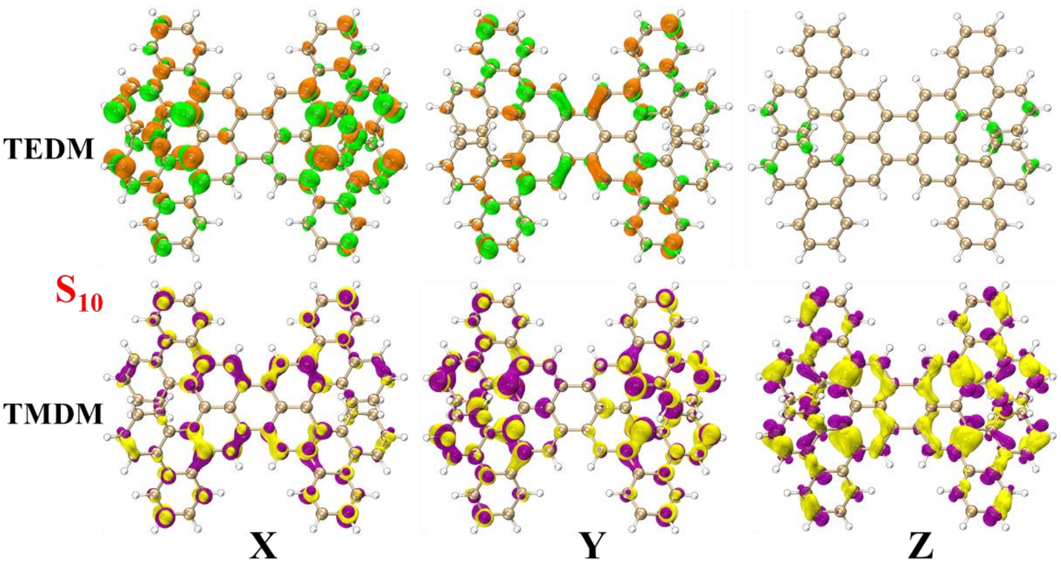

Figure S16. TEDM and TMDM of 1- (M, M) in S_{10} excited state.

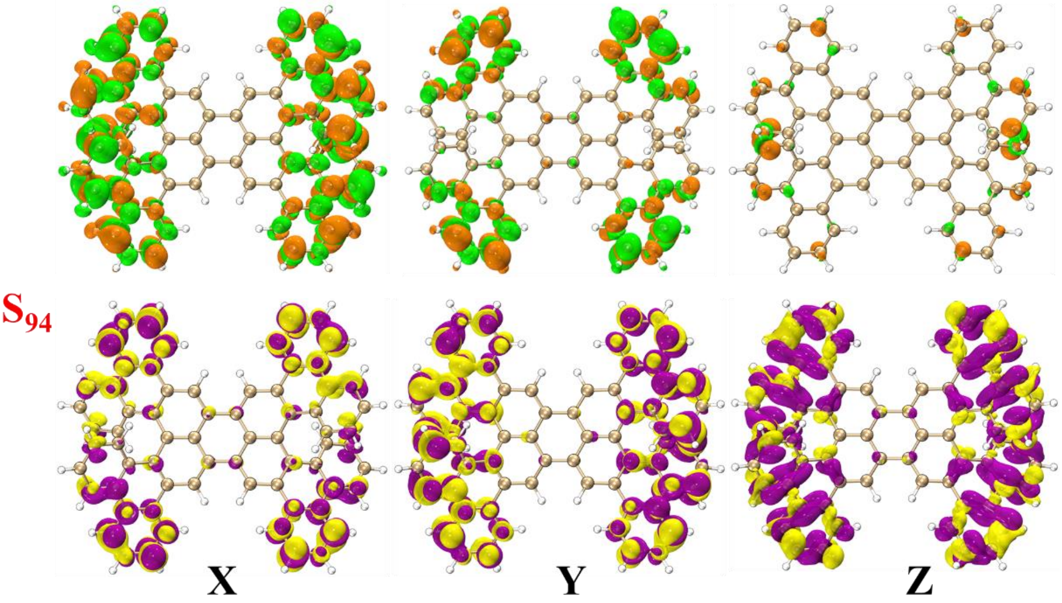

Figure S17. TEDM and TMDM of 1- (M, M) in S_{94} excited state.
